# Biddle's Materia Medica. Eighth Edition

**Published:** 1878-01

**Authors:** 


*Materia Medica, for the use of Students.*—By John B. Biddle, M. D., Prof. of Materia Medica and General Therapeutics, in the Jefferson Medical College, Etc., Etc. Eighth Edition. Revised and Enlarged. Publishers: Lindsay and Blakiston, Philadelphia. 1878,

Another New Edition of this well known treatise, is an evidence of its popularity in the Medical Profession. For years past it has been the recognized text book in a large number of the most respectable of the Medical Colleges, and each revision adds to its value and increases its usefulness to the student of medicine. The preceding edition was exhausted within little more than a year after its issue, and in the present edition considerable has been rewritten and many new articles added. The illustrations, of which this work contains quite a number, represent the most important of the plants valued for their medicinal qualities, as well as instruments employed for the atomization of liquids in pneumatic aspiration, a comparatively recent process for the treatment of thoracic diseases, and which is also available in the transfusion of blood.

Dental as well as Medical Students will derive great benefit from this work on Materia Medica and Therapeutics. The type with which the work is printed are large and distinct, and the style of binding, &c., is creditable to the well known publishers of medical and dental works.

